# An Ultra-Short Baseline Underwater Positioning System with Kalman Filtering

**DOI:** 10.3390/s21010143

**Published:** 2020-12-28

**Authors:** Qinghua Luo, Xiaozhen Yan, Chunyu Ju, Yunsai Chen, Zhenhua Luo

**Affiliations:** 1 School of Information Science and Engineering, Harbin Institute of Technology at Weihai, Weihai 264209, China; luoqinghua80@hit.edu.cn (Q.L.); 19s130296@stu.hit.edu.cn (C.J.); 2 Automatic Test and Control Institute, Shandong Institute of Shipbuilding Technology, Weihai 264209, China; 3 Department of Technology, New Beiyang Information Technology Co., Ltd., Weihai 264203, China; 4 China National Deep Sea Center, Qingdao 266237, China; cys@ndsc.org.cn; 5 School of Water Energy and Environment, Cranfield University, Cranfield MK43 0AL, UK; Z.Luo@cranfield.ac.uk

**Keywords:** acoustic signal detection, adaptive filters, Kalman filters, signal denoising

## Abstract

The ultra-short baseline underwater positioning is one of the most widely applied methods in underwater positioning and navigation due to its simplicity, efficiency, low cost, and accuracy. However, there exists environmental noise, which has negative impacts on the positioning accuracy during the ultra-short baseline (USBL) positioning process, which results in a large positioning error. The positioning result may lead to wrong decision-making in the latter processing. So, it is necessary to consider the error sources, and take effective measurements to minimize the negative impact of the noise. In our work, we propose a USBL positioning system with Kalman filtering to improve the positioning accuracy. In this system, we first explore a new kind of element array to accurately capture the acoustic signals from the object. We then organically combine the Kalman filters with the array elements to filter the acoustic signals, using the minimum mean-square error rule to obtain accurate acoustic signals. We got the high-precision phase difference information based on the non-equidistant quaternary original array and the phase difference acquisition mechanism. Finally, on account of the obtained accurate phase difference information and position calculation, we determined the coordinates of the underwater target. Comprehensive evaluation results demonstrate that our proposed USBL positioning method based on the Kalman filter algorithm can effectively enhance the positioning accuracy.

## 1. Introduction

Position information determines the accuracy and efficiency of underwater operations and exploration works, especially for deep-water operations. So, positioning services are playing an increasingly important role in the development of marine science and technologies [1,2]. Many scholars and researchers have a great interest in underwater positioning in many fields, including undersea target tracking, marine resource development, and underwater vehicle positioning and navigation [2,3,4].

According to the baseline length of the acoustic positioning system, the positioning systems can be classified traditionally into three types [1,2,3]: the long baseline (LBL) positioning system, the short baseline (SBL) positioning system, and the ultra-short baseline (USBL) positioning system. Relative to the other two types, the USBL possesses the advantages of its simplicity, efficiency, and accuracy [5,6,7]. However, due to the negative impact of some uncertain factors [1,4,5,6,7], including multi-path propagation of acoustic signals, environmental interference, and installation error during the positioning procedure, these uncertain factors lead to poor positioning results, which cannot meet the requirements of many applications, or even worse, it may provide a wrong reference value for the latter processing methods. To improve the positioning accuracy, many researchers and scholars presented numerous works [1,6,7,8,9,10,11,12,13,14,15,16,17,18,19,20,21,22,23]. The authors in [2,3,4,5,6,7,8,9] analyzed the errors and faults during underwater positioning; it took measurements to restrain the error. The authors in [1,10,11] utilized filters or multiple interacting models to improve the positioning accuracy. References [12,13,14,15,16] proposed some new kinds of positioning methods to improve the positioning accuracy. References [17,18] focused on different types of element arrays to enhance the underwater positioning system. In [19,20,21], the jumping point problem and large delay difference between the positioning data were dealt with. References [22,23,24] proposed some integrated navigation methods based on acoustic localization, which played a critical role in improving the positioning accuracy. However, due to the limitations of the method, including the enormous scope of system redundancy information, the improvement was not promising.

In this paper, considering the measurement error sources and their influence mechanism on the positioning results, we propose a USBL positioning system based on the Kalman filtering algorithm to improve the positioning accuracy. In summary, the main contributions of this paper are as follows.

(1) Considering the error source and its impact on the positioning results, we present the Kalman filter-based, non-equidistant quaternary array. We organically combine the Kalman filtering and array element to accurately capture the acoustic signals.
(2) During the USBL positioning process, we utilize an array element and a corresponding processing method to eliminate the ambiguity problem of a phase difference, which can improve the accuracy of our proposed USBL positioning system.
(3) Based on the capture of the acoustic signal and calculation of the phase difference, we present an ultra-short baseline underwater positioning system with Kalman filtering to enhance the positioning accuracy.

The structure of the paper is organized as follows: Section 2 reviews the related works. Section 3 puts forward the proposed USBL positioning method based on the Kalman filtering acoustic signals, to obtain accurate phase differences. Section 4 verifies and evaluates the performance of the proposed positioning algorithm. Finally, we conclude the research work.

## 2. Related Works

Many researchers have been focusing on the array elements designing and positioning problems of USBL positioning systems in recent years. They proposed various types of array element types [1,2,15,16,17,18]. Many positioning methods also have been presented to improve the positioning accuracy [4,5,6,7,8,9,10,11,12,13,14,15,16,19,20,21,22,23,24].

### 2.1. The Array Types

According to the deployment of primitive elements, there are numerous array types, including the traditional triangular matrix, the orthogonal, and non-orthogonal element arrays. To improve the calibration efficiency and enhance the adaptability of the calibration, the authors in [1] proposed multiple interacting models and unscented Kalman filter calibration to gain a faster convergence rate based on a traditional array. Reference [2] studied different installation errors in the USBL transceiver array and proposed a dynamic calibration algorithm based on an incremental iteration. Reference [15] designed a complete traditional USBL positioning system. The literature also presented an orthogonal eight elements array and an orthogonal quaternary array. Compared with the traditional array, they could improve the positioning accuracy 8-fold. Underwater positioning provides a critical service for underwater acoustic (UWA) networks [16,17]; Reference [18] employed the paradigm of the software-defined networking (SDN) technology and proposed an SDN-based underwater cooperative searching framework for autonomous underwater vehicle (AUV)-based underwater wireless networks (UWNs). The simulation results demonstrated the proposed scheme’s validation, but the experimental system was not deployed and evaluated. In [19,20], a quaternary array with an unequal spacing was proposed. This method, with the same positioning accuracy as the orthogonal array composed of eight elements, can reduce the number of primitive elements and increase the utilization of primitive elements. From the view of signal noise reduction, most systems took advantage of the adaptive filtering algorithm or adaptive residual optimization algorithm to filter signals to obtain the signal phase difference [4,10], but the result was not promising. Reference [21] proposed a novel non-equidistant, quaternary array. This method could improve the positioning accuracy 8-fold, with much fewer array elements. However, they did not consider the ambiguity problem of a phase difference. In summary, the research works focused on developing different kinds of arrays to capture the acoustic signals from the target. We should consider the signal error and ambiguity problem of a phase for high position accuracy.

### 2.2. Positioning Methods

Wang et al. proposed a robust Student’s *t*-based Kalman filter for the strap-down inertial navigation system (SINS) and the ultra-short baseline (USBL) integration system [3], which was utilized to suppress the measurement uncertainty induced by the acoustic outliers. Reference [4] established a USBL observation model and a grid SINS/Doppler Velocity Log (DVL)/USBL-integrated navigation system to restrain the SINS errors. When the marine environment changed in the shallow sea area, the propagation speed model needed to be corrected. The authors in [5] proposed a combined ray-tracing method to determine whether to use the constant acoustic speed ray-tracing method or the equal gradient ray-tracing method. References [6,7] considered the installation error and multi-path error during underwater positioning and proposed calibration and reduction methods to enhance the positioning method. Reference [8] analyzed the USBL sea-trial calibration and its application to a real diving environment; the experimental results illustrated that the positioning performance is consistent with the calibration results. Reference [9] presented a combined ray-tracing method to reduce the error of slant distance. In [10], an adaptive Kalman filter to improve the positioning accuracy was adopted. References [11,12] designed a new SINS/USBL-coupled, integrated navigation algorithm based on the phase difference measurement. In [13,14], they proposed some underwater positioning techniques and demonstrated how to apply them to beam formation in multi-user underwater acoustic communications. Reference [15] investigated the impact of different array designs on the accuracy of object location. They proposed the designing method based on the Johnson solids and devised a specific algorithm to evaluate the positioning performance. By employing the paradigm of SDN technology, Reference [18] proposed an SDN-based underwater cooperative searching framework for the AUV-based UWNs. Considering the location solution, in [22] the target depth information, to remove the influence of a large delay difference on a specified axis, was used. Reference [23] put forward an identification and elimination method of the outlier point in the positioning data during processing. However, because of the more restrictive scope of the above two applications, the improvement was limited. In [24], a Student’s *t*-based Kalman filter, to process the uncertainty of the SINS/GPS integration application, was proposed. For indoor tracking of dynamic positioning, the authors in [25] presented a scheme based on the distributed, multi-sensor data-fusion method to improve positioning accuracy. Reference [26] presented an underwater positioning algorithm based on a SINS/LBL integrated system. The algorithm is just applied to the LBL positioning environment. In brief, there are many uncertain factors during underwater positioning computation. We should pay special attention to the error resources and the corresponding processing strategies to improve the underwater positioning performance.

In this study, we present a USBL positioning method based on Kalman filtering to improve the positioning accuracy. Different from other research works, we explore the error resources appearing during the phase difference acquisition and positioning process. We utilize Kalman filters combined with each array element to filter the received acoustic signals, to obtain accurate signals with the minimum mean-square error rule as the best estimation criterion. As the filtering of the received signals can be performed in parallel, the phase difference is gained accurately. The Kalman filtering-based underwater positioning system has the advantage of a high accuracy and low complexity.

## 3. The USBL Positioning System Based on Kalman Filtering

In the following content, we first introduced the framework of our proposed positioning method. We then describe the detailed function and specific implementation of each sub-module.

### 3.1. The Framework of the USBL Positioning Method Based on Kalman Filtering

**Figure 1:**
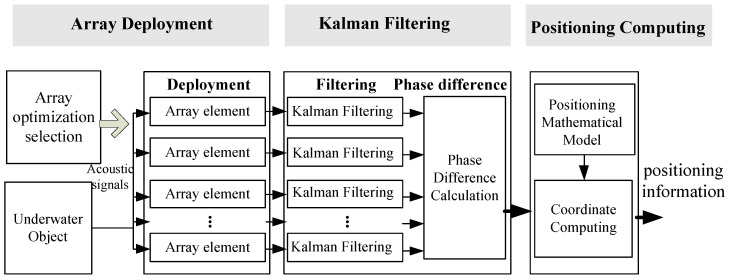
The framework of the ultra-short baseline (USBL) positioning system based on Kalman filtering.

We show the framework of the underwater positioning system as Figure 1. It is composed of three main components: “Array Deployment”, “Kalman Filtering”, and “Positioning Computing”.

#### 3.1.1. Array Deployment

In the framework, we can adopt any type of element array. For a higher accuracy, in this work, we adopted a new array: the non-equidistant, quaternary array. It reuses an array element three times and utilizes a specific processing method, which can improve the positioning accuracy. The array elements received the acoustic signals from the target. Since there are position differences between the primitive elements in the array, each of the primitive element will receive the same acoustic signal from the target with different delays. So, the received acoustic signals of these primitive elements have phase differences. The purpose of the array setting is to receive the acoustic signal, obtain the phase difference, and facilitate the next coordinate calculation.

#### 3.1.2. Kalman Filtering

The Kalman filtering algorithm is exploited with an array to reduce the signal noise; i.e., to filter out the Gaussian white noise in the received acoustic signal and improve the signal quality. We can obtain the phase differences between different received acoustic signals to provide prior information for the next step by setting an adjustable threshold.

#### 3.1.3. Positioning Computing 

After obtaining the phase difference with high accuracy between the received signals, we obtain the positioning result through positioning computing by a mathematical model.

### 3.2. Array Deployment

In this section, we first illustrate the traditional array and its characteristics. To overcome its limitation, we adopt a new type of array. We then describe the non-equidistance, quaternary array.

#### 3.2.1. Traditional Array

A traditional ultra-short baseline (USBL) usually utilizes a triangular matrix or a cross orthogonality matrix, whose baseline size is smaller than a half-wavelength of the transmitted signal. The USBL is applied widely to various underwater positioning systems due to its small size and easy installation. Furthermore, the USBL positioning system utilizes the phase difference between the received signals of each primitive element to calculate the target orientation and distance.

**Figure 2:**
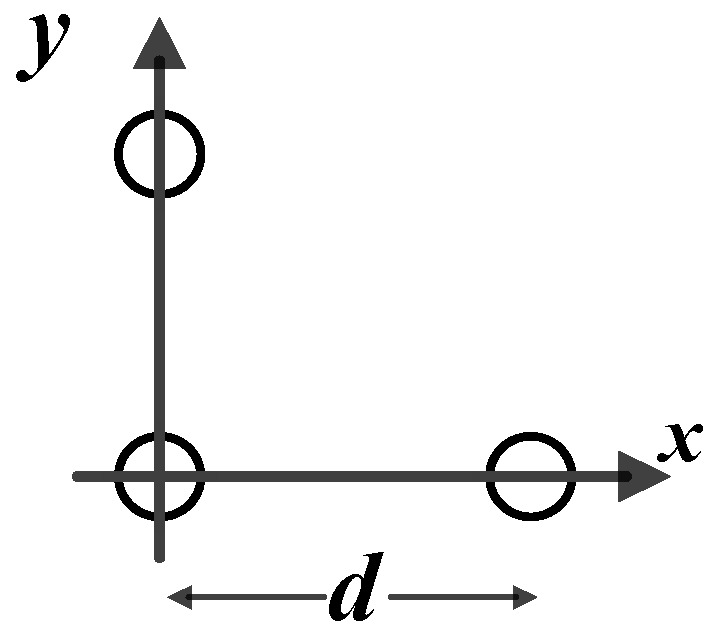
A traditional USBL orthogonal array diagram.

A traditional USBL orthogonal array is demonstrated in Figure 2. The three array elements are deployed in an isosceles, right-angled triangle, and the array element distance is d. Usually d is smaller than λ, where λ = 80 mm. λ is the wavelength of the acoustic wave to avoid ambiguity in phase measurement. If we use such a small array size alone, it will be difficult to locate the remote target to achieve the positioning accuracy of 0.5%, while the positioning accuracy of a traditional USBL is about 3%. From the analysis of principle, increasing the baseline length of the array can reduce the positioning errors. By reducing the operating frequency band of the system and increasing the dimension between array elements, high accuracy positioning can be achieved. The multi-array element processing technique is another effective way to improve system positioning accuracy.

#### 3.2.2. Non-Equidistant, Quaternary Array

**Figure 3:**
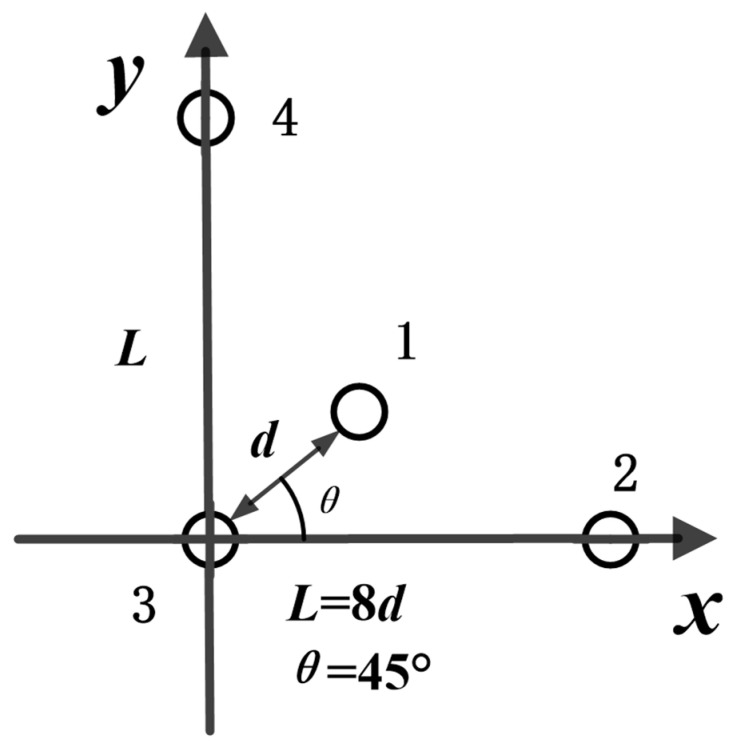
The non-equidistant, quaternary array diagram.

In our study, we deployed the non-equidistance, quaternary array of the proposed USBL positioning system. It is demonstrated in Figure 3. The array element spacing is d, and the parameter settings are the same as the traditional USBL orthogonal array. The angle between the *x*-axis and Element 1 is equal to 45°. The distance L between Element 3 and Element 2 is 8-fold that of d; the same to distance L exist between Element 3 and Element 4. To solve the ambiguity phenomenon of the phase difference between Array Element 2 and Array Element 3, and between Array Element 3 and Array Element 4, we utilized the projection of the signal received by Element 1 and Element 3 on the *x*-axes and the *y*-axes, respectively. The advantage of the new array is that it could reuse the signals received by Array Element 3 three times, reducing the design of additional, redundant array elements.

### 3.3. Signal Noise Reduction

Noise can cause significant disturbance to the underwater object’s positioning calculation. So, it is necessary to conduct noise reductions in the underwater acoustic signal to achieve a higher accuracy of the target’s positioning. However, the propagation of the acoustic signal in the water propagation is affected by the temperature, waves, and internal waves. It will make noise distribution random and irregular. We chose the least-square error rule as the best estimation criterion for the noise reduction of the signals received to reduce the noise interference and improve the positioning accuracy. According to this idea, the paper utilizes the Kalman filtering algorithm to process the obtained acoustic signals based on the minimum mean-square error estimation.

Kalman filtering estimates the system state from the sequences of uncertain observations using the predict–update cycle. First, the next system state and its uncertainty are predicted by an existing physical model and a statistical model, which describes any uncertain factors, including the process noise. This prediction is updated using the procedure observation and the difference between the prediction value and the observation value. Once the updated estimation is done, we can estimate a new predictive value.

The Kalman filtering algorithm requires only the system estimation data of the previous moment and the current measurement data in each operation; the algorithm is simple and easy to implement. It also has a wide range of applications in the field of underwater navigation. In this study, we utilize the Kalman filter algorithm to smooth the acoustic signal. The specific method we used is as follows.

In the underwater positioning array, shown in Figure 3, the array elements receive acoustic signals from the underwater target. We sample the received signals and get a sequence *S* = {*s*_1_, *s*_2_, *s*_3_, …, *s_k_*, …, *s_n_*}, where *k* is the *k*th moment stamp, *n* is the total number of sampling, and 1 ≤ *k* ≤ *n*.

(1) $$u_{k}=s_{k}/(\max(S)-\min(S))$$

To reduce the negative impact of noise, we utilize a Kalman filter to filter the signal {$u_{k}$} using Equations (2) and (3).

(2) $$X_{k}=H*X_{k-1}+G*W_{k-1}$$

(3) $$u_{k}=H*X_{k}$$

Here, *X* = {*X*_1_, *X*_2_, *X*_3_, …, *X_k_*, …, *X_n_*} is the state information of the system. *H* is the transition matrix (we set it to be a unit matrix), and *G* denotes the gain matrix; *W_k−_*_1_ is the system noise at the moment *k* (we treat it as Gaussian white noise); {*u_k_*} presents the system observation information of the system; *X_k_* denotes the state value of the system at moment *k*; and *u* = {*u*_1_, *u*_2_, *u*_3_, …, *u_k_*, …, *u_n_*} is the observation signal of the system.

There are two procedures durin Kalman filtering: estimation and correction. In the estimation stage, we estimate the system state ${\hat{X}}_{k}$ at the moment *k* by Equation (4), and the prior state estimate ${\hat{X}}_{k}^{-}$ is estimated recursively at the *k*th moment by state ${\hat{X}}_{k-1}^{-}$ at moment *k*−1, where there is no control input.

(4) $${\hat{X}}_{k}^{-}=A{\hat{X}}_{k-1}^{-}$$

Equation (5) predicts the mean-squared error $P_{k}^{-}$ of the system, and the prior covariance estimation *P_k_*_−1_ is obtained recursively at the *k*th moment through the posterior covariance $P_{k}^{-}$ at the previous time.

(5) $$P_{k}^{-}=AP_{k-1}A^{T}+Q$$

Equation (6) calculates the Kalman gain of the system, and the system Kalman gain *K_k_* is calculated at the *k*th moment through the covariance estimation $P_{k}^{-}$ at moment *k*.

(6) $$K_{k}=P_{k}^{-}H^{T}{\left(HP_{k}^{-}H^{T}+R\right)}^{-1}$$

Equation (7) calculates the estimated value of the system state, and the system state value $X_{k}$ is calculated at the *k*th moment through the prior state estimation at the *k*th moment.

(7) $$X_{k}=H*X_{k-1}+G*W_{k-1}$$

We estimate the posterior error covariance $P_{k}$ of the system by Equation (8). Here, *K_k_* is the system gain and $P_{k}^{-}$ denotes the prior error covariance.

(8) $$P_{k}=(I-K_{k}H)P_{k}^{-}$$

Finally, we can obtain the processing result ${\hat{u}}_{k}$ by Equation (9).

(9) $${\hat{u}}_{k}={\hat{X}}_{k}$$

In these analyses, $\hat{X}$ = {${\hat{X}}_{1}$, ${\hat{X}}_{2}$, ${\hat{X}}_{3}$, …, ${\hat{X}}_{k}$, …, ${\hat{X}}_{n}$} is the state information of the system and $\hat{X}$ is the state variable of the system at moment *k*. ${\hat{X}}^{-}$ = {${\hat{X}}_{1}^{-}$, ${\hat{X}}_{2}^{-}$, ${\hat{X}}_{3}^{-}$, …, ${\hat{X}}_{k}^{-}$, …, ${\hat{X}}_{n}^{-}$} is the prior state estimation of the system. ${\hat{X}}_{k}^{-}$ is the prior estimation of the state variable at the *k*th moment, obtained from the state variable at moment *k*−1. *A* = 1 is the state transform coefficient acting on ${\hat{X}}_{k-1}^{-}$. *H* = 1 is the observation model matrix, which maps the real state space into observation space. $P^{-}$= {${\hat{P}}_{1}^{-}$, $P_{2}^{-}$, $P_{3}^{-}$, …, $P_{k}^{-}$, …, $P_{n}^{-}$} is a 1 × *n* prior estimation error covariance matrix; *P* = {*P*_1,_ *P*_2_, *P*_3_, …, *P_k_*, …, *P_n_*} denotes a 1 × *n* posterior estimation error covariance matrix; *Q* = 0.1 represents the process noise covariance coefficient; *R* = 0.25 is the process noise covariance coefficient; the value of *I* is set to be 1; and *K* denotes the Kalman gain or mixing factor, calculated from the specific data. Its role is to minimize the posterior estimation error covariance. *û* = {*û*_1_, *û*_2_, *û*_3_, …, *û_k_*, …, *û_n_*} denotes the filtered value of the acoustic signal, and *û_k_* denotes the filtered value of the acoustic signal at moment *k*.

The above process is repeated recursively to implement the filtering processing of the acoustic signal received. We transform the original signal *u* = {*u*_1_, *u*_2_, *u*_3_, …, *u_k_*, …, *u_n_*} into a new signal *û* = {*û*_1_, *û*_2_, *û*_3_, …, *û_k_*, …, *û_n_*}. Similarly, we can get the processed signals *û_x_*, *û_y_*, and *û_o_* from the *x*-axis array element, the *y*-axis array element, and the original array element, respectively. The filtering method adjusts the relevant parameters by an estimation error condition and realizes higher-quality signal filtering.

### 3.4. Positioning Computing

#### 3.4.1. Positioning Principle

Firstly, we introduce the traditional principle of phase difference positioning. For the single-frequency CW signal, the phase information is the most commonly used for the ultra-short baseline positioning system. The phase difference between the received signals is measured to solve the positioning problem. We utilize different frequencies to distinguish the target response signals in multi-target situations. The following describes the principle of target location using the phase difference between single-frequency CW signals.

**Figure 4:**
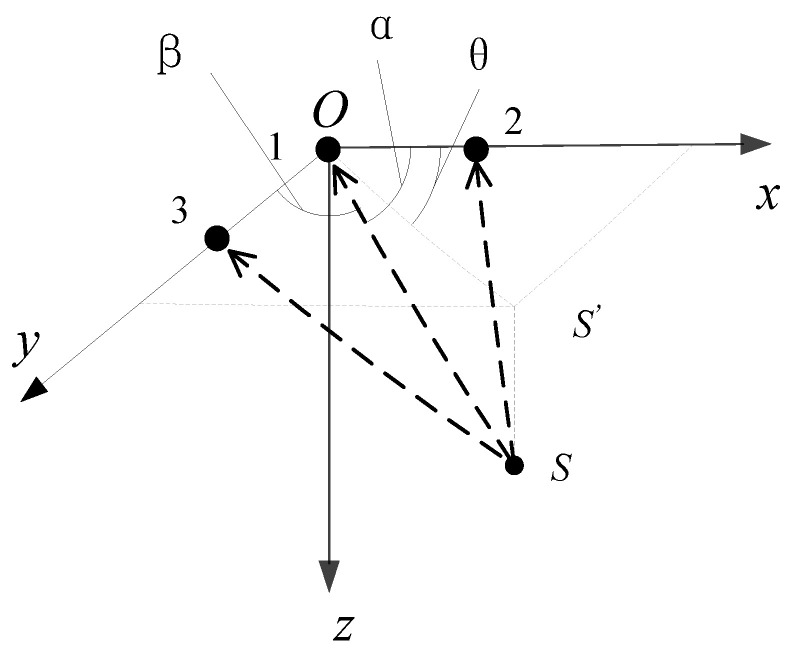
The principle of positioning.

We performed underwater positioning, as shown in Figure 4. In the specific coordinate system, we need to determine the coordinate (*x*, *y*, *z*) of target *S*. We deployed two orthogonal linear arrays on the *x*-axis and *y*-axis, respectively, and the array center is the origin of the coordinates.

The target radius is OS, and its direction cosine is

(10) cosα=x/R

(11) cosβ=y/R

(12) $$R=\sqrt{x^{2}+y^{2}+z^{2}}$$

Here, α represents the radius OS and the *x*-axis angle; β denotes the radius OS and y-axis angle; *R* is the target slant distance; and S’ is the projection of S on the “*x*o*y*” plane, and the angle *θ* between it and the *x*-axis is the target horizontal azimuth. We can obtain the angle *θ*:

(13) $$\cos\alpha =x/R\theta =tg^{-1}\left(y/x\right)=tg^{-1}\left(\cos\beta /\cos\alpha \right)$$

(14) $$r=\sqrt{x^{2}+y^{2}}$$

(15) $$z=\sqrt{R^{2}-r^{2}}$$

Here, *r* is the target horizontal slant distance and *z* is the target depth. We can determine it through a depth measurement sensor.

Equations (10)–(15) are the basic formulas for positioning calculation. The above equations can work out the target position parameters. Considering the propagation of the acoustic signals between the element array and object, and the propagation differences between the elements, we can get the equations approximately.

(16) ϕ=(2πdcosα)/λ

(17) ψ=(2πdcosβ)/λ

Here, ϕ is the phase difference of the received signal from the adjacent array element on the *x*-axis; λ represents the wavelength of the acoustic signal; and ψ denotes the phase difference of the received signal from the adjacent array element on the *y*-axis.

With Equations (10) and (11), we can obtain Equations (18) and (19). We determine the coordinate (*x*, *y*, *z*) of the target relative to the USBL’s position.

(18) x=(λϕR)/2πd

(19) y=(λψR)/2πd

In the formulas above, *R* = *c* × ∆*t*/2, *c* is the acoustic velocity in water and ∆*t* is the time difference from the transmission to the receiver. So, the distance *R* is equal to *c* × ∆*t*/2, so the actual measurements are ϕ, ψ, *c*, and ∆*t*.

#### 3.4.2. Ambiguity Problem Solution for the Phase Difference

During the actual measurement, the acoustic velocity *c* is the same. The error ∆*t* is negligible, and the accuracy of the slope distance *R* estimated by the response ranging method is high enough. The estimation accuracy of *R* is called the vertical estimation accuracy. The estimation accuracy of *x* and *y* is called the horizontal coordinate estimation accuracy. It follows from Equations (17)–(19) that they mainly depend on the measurement accuracy of the phase differences ϕ and ψ.

After the noise reduction in Section 3.3, we analyze the signals *û_x_*, *û_y_*, and *û_o_* to obtain the phase difference between the signals. Then we plug the phase differences *ϕ* and *ψ* into Equations (18) and (19) to get the coordinates (*x*, *y*) of the target; the values of *x* and *y* are then plugged into Equation (12). We calculate the depth *z* of the target to achieve the positioning calculation.

However, there exist ambiguity issues of a phase difference. We solve this problem as follows. According to Equations (18) and (19), considering the distance between Element 1 and Element 3, and the distance between Element 2 and Element 3 on the *x*-axis, Equations (20) and (21) are obtained, respectively.

(20) $$x_{d}=(\lambda \phi_{13}R)/2\pi d$$

(21) $$x_{L}=(\lambda \phi_{23}R)/2\pi L$$

Here, $x_{d}$ and $x_{L}$ are the projections of the distance *R* on the *x*-axis and in the Array Element 1 direction, respectively.

In this research work, *ϕ*_13_ is the phase difference between Element 1 and Element 3. There is no phase ambiguity. So, we can utilize it to confirm there is phase ambiguity in *ϕ*_32_ and *ϕ*_34_ for a high positioning accuracy or not. The signal delay differences between all azimuths received by Array Element 1 and Array Element 3 are both equal to *τ*_13_ and less than T/2. So, the phase difference *ϕ*_23_ is equal to 8*ϕ*_13_. When |*ϕ*_13_| is greater than 22.5°, *ϕ*_23_ will be located in the multi-valued interval, and the difference between the measured value *ϕ*_23_ and the true value is one or several cycles; this as long as *ϕ*_23_ is added or subtracted by an integer multiple of 360° and compared with 8-fold of *ϕ*_13_. If the phase difference is less than one cycle, we can determine that the *ϕ*_23_ is correct. This step will solve the phase difference multi-valued fuzzy of a large array.

Next, we consider the positioning accuracy of the new array. *ϕ_ij_* and *ψ_ij_* have the same phase difference measurement accuracy; i.e., ∆*ϕ*_13_ = ∆*ϕ*_23_ = ∆*ϕ* when the background noise of the array elements is independent.

We regard Equation (20) as the positioning formula on the *x*-axis of the traditional USBL array. We treat Equation (21) as the improved positioning formula on the *x*-axis of the USBL array. Without considering the measurement errors of the slope *R* and the sound speed c, we differentiate both sides of Equations (20) and (21) respectively to get the influence factor of *x_d_* and *x_L_*:

(22) $$\Delta x_{d}=\Delta \phi_{13}\left(\lambda R\right)/\left(2\pi d\right)=\Delta \phi \left(\lambda R\right)/\left(2\pi d\right)$$

(23) $$\Delta x_{L}=\Delta \phi_{23}\left(\lambda R\right)/\left(2\pi L\right)=\Delta \phi \left(\lambda R\right)/\left(2\pi L\right)$$

Since *L* = 8 *d*, the array element with spacing *L* can improve the positioning accuracy 8-fold from Equations (20) and (21). The array element with spacing *L* can solve the positioning accuracy problem to achieve a high-precision phase measurement positioning based on the array element with a spacing *d*, solving the multi-valued phase difference measurement blurring.

From the illustration above, we analyzed the computation complexity of the proposed Kalman filtering-based underwater positioning method. Suppose the number of positioning points is *M*, the sample point number of the acoustic signal is *n*. The computational complexity is O(*Mn*).

## 4. Performance Evaluation and Analysis

In this subsection, we evaluate and analyze the proposed method, which is based on the Kalman filtering algorithm for the acquisition of the phase difference. We assessed the performance of the proposed system. We also compared it with the related methods to verify its feasibility and validation.

Firstly, we describe the setup of the simulation environment. We then analyze the influence of sampling frequency on positioning accuracy in different noise environments. Finally, we evaluate the performance of the proposed method and make a comparison with other related methods.

### 4.1. Evaluation Environment Setup

#### 4.1.1. Evaluation Design

We first set up the simulative signal and then calculate the expected delay value through the pre-set phase difference between the signals of the array elements. We then utilize the dense sampling interval to approximate the real-time delay of a phase difference between the acoustic signals. We adopt the initial phase of the signal to compensate for the non-integer, multiple-sampling interval delay values; this will bring it as closely as possible to the actual situation.

#### 4.1.2. Parameters Setting

We obtain the values by calculating that ∆*t*_12_ = 6.134 × 10^−6^ s (the delay between Array Element 3 and Array Element 2), and ∆*t*_13_ = 1.463 × 10^−5^ s (the delay between Array Element 3 and Array Element 4). The original signal is CW = *A* × cos (2 × π × *f*_0_ × *t*−30 × π/180). The initial phase is −π/6, the amplitude is *A* (*A* = 0.5), and the angular frequency is 2π*f*_0_ (*f*_0_ = 1350 Hz). We illustrate the simulation conditions in Table 1.

**Figure 5:**
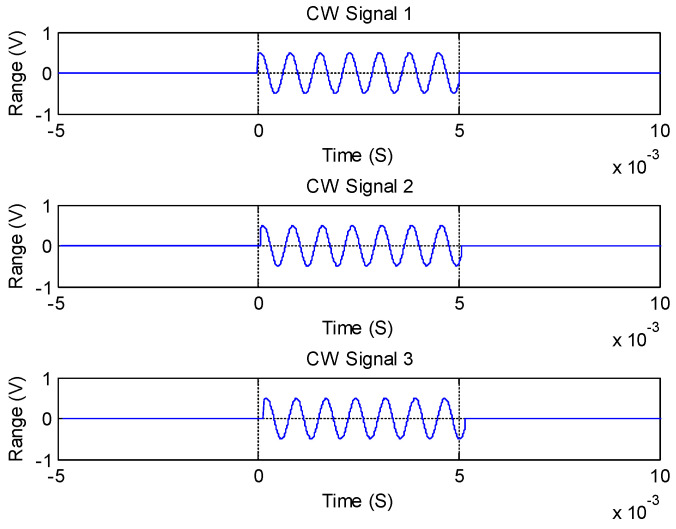
The original three-channel original input signal waveform diagram.

**Figure 6:**
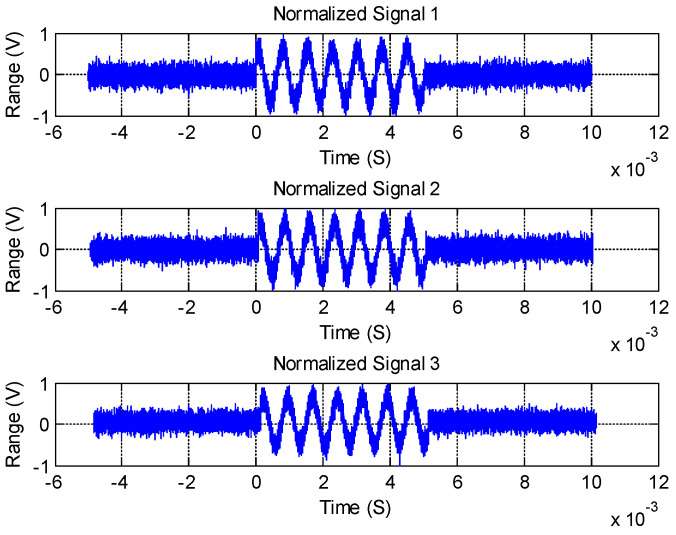
The three-way received signal waveform diagram.

We show the original signals in Figure 5, which are received by Element 3, Element 2, and Element 4, respectively. We determined the signal amplitude by the preset *SNR* and the normalized noise. Taking the target with a frequency of 1.35 kHz as an example, we show the waveform of the three-element receiving signal generated by the simulation in Figure 6. The *SNR* was 20 dB in these processes. We set the pulse width to 5 ms, and the sampling frequency was 20 kHz.

Figure 6 shows that the general waveform profile is still visible after the signal plus noise; but, the specific parameters are ambiguous, which will cause a large interference regarding the positioning accuracy.

#### 4.1.3. Evaluation Metrics

During the evaluation of positioning accuracy, since the target coordinate positions (*x*, *y*) are obtained by measuring the phase differences *ϕ* and ψ the errors of *x* and *y* are introduced by the measurement errors of the phase differences *ϕ* and ψ. The errors are the same, and the accuracy of the results, using the following simulation, is only through the errors in *x* and *y*. Hence, the positioning accuracy is calculated as *σ_x_* and *σ_y_*. *σ_x_* and *σ_y_* can be calculated by Equations (24) and (25), respectively.

(24) $$\sigma_{x}=\sqrt{\left[\sum_{i=1}^{N}{\left(x_{i}-x_{0}\right)}^{2}\right]/N}●\left(1000/R\right)$$

(25) $$\sigma_{y}=\sqrt{\left[\sum_{i=1}^{N}{\left(y_{i}-y_{0}\right)}^{2}\right]/N}●\left(1000/R\right)$$

In Equations (24) and (25), (*x_i_*, *y_i_*) is the positioning result, and (*x*_0_, *y*_0_) denotes the reference coordinate. *R* is the target slant range, and its measurement error is not considered.

#### 4.1.4. Reference Methods

In this paper, we adopted three reference methods. They are the adaptive filtering [10], adaptive residuals filtering [4], and new four-element methods [19]. In the adaptive filtering method [10], a least mean-square (LMS) algorithm is utilized to update the adaptive parameters to filter noise. Due to the inaccurate estimation of the gradient value, the adaptive filtering is with noise. To improve the position accuracy, the adaptive residuals filtering algorithm [4] adopts adaptive residuals correction strategy, and the convergence rate is enhanced. The new four-element method adopts the new kind of array. However, the phase difference is gained in a traditional way. The improvement is not promising.

### 4.2. Impact Analysis of Sampling Frequency on Positioning Accuracy

With the same signal-to-noise ratio (*SNR*), different sampling frequencies have different effects on noise reduction during signal processing. In general, the higher the sampling frequency, the better the noise reduction it makes. However, the computational complexity and computational time will increase when increasing the sampling frequency, which will affect the real-time performance of the positioning method. Therefore, a suitable sampling frequency needs to be determined to satisfy the positioning accuracy requirement of making the computing time as small as possible. We consider the location accuracy and the corresponding running time when the signals are processed at different sampling frequencies. So, we can determine a suitable sampling frequency to meet the requirement of the application system.

**Figure 7:**
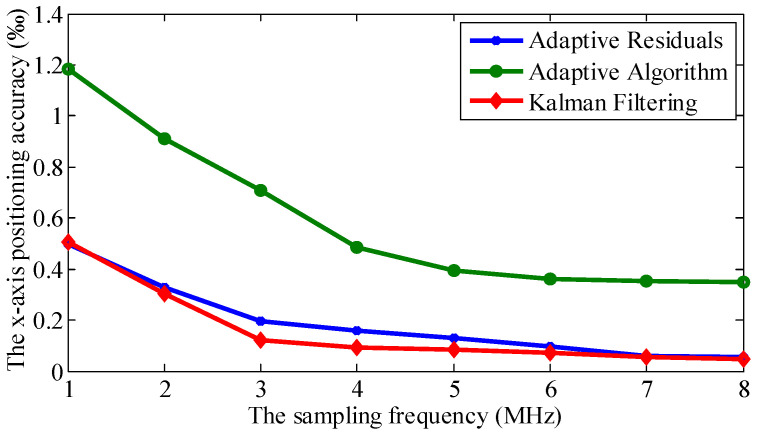
Comparison of the positioning accuracies under different sampling frequencies.

We set the value of the *SNR* to 20 dB. We adjusted the sampling frequency from 1 MHz to 8 MHz with an interval of 1 MHz. The signals were denoised by an adaptive algorithm [10], an adaptive residuals algorithm [4], and Kalman filtering at the eight different sampling frequencies, respectively. We show the *x*-axis positioning accuracy of the three methods with different sample frequencies in Figure 7; the y-axis positioning accuracies of the three methods are of the same level.

Figure 7 shows that the proposed Kalman filtering method has a lower positioning error than the other two methods under the same *SNR*. The adaptive filtering algorithm replaces the mean squared error directly with the single-sampled data error square. It causes an inaccurate gradient estimate in each iteration of the adaptive process. It means that the whole adaptive process is noisy, and it will not strictly move along the real fastest path of descent on the performance surface. The residual adaptive optimization algorithm increases the weighted coefficient based on the adaptive filtering algorithm, making the residual weight in the iteration processes the largest one. Although it accelerates the convergence speed and reduces the adaptive weight noise, the noise cannot be ignored. Based on the previously estimated value and the recently observed data, the Kalman filtering algorithm takes the minimum mean-square error as the estimation criterion to estimate the current signal value. Through denoising the observation signal, we obtain the estimated signal with the smallest error. Figure 7 describes that when the sampling frequency is greater than 2 MHz, the positioning accuracy with the Kalman filter is 3- to 6-fold higher than that of the adaptive filtering result, and 1.2- to 1.6-fold the accuracy of the adaptive residual filtering result. When the sampling frequency is more than 2 MHz, the positioning accuracy is higher than 4‰. It meets the requirement of a general application system.

What is more, we find that the running time has a positive relationship with the sampling frequency throughout the experiment. Considering the conditions to meet a high accuracy and less operating time, we can state that the sampling frequency of 2 MHz is more appropriate.

### 4.3. Positioning Accuracy Evaluation with Traditional Array

**Figure 8:**
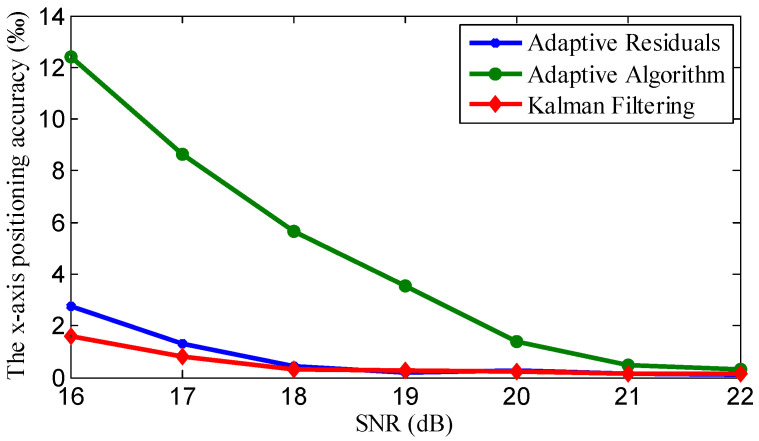
Positioning accuracy comparison of different methods with different SNR values.

With the traditional array, we evaluate the positioning accuracy of different processing methods. We deployed the element array as in Figure 2, and the parameters’ settings are shown in Table 2. We compared three processing methods: adaptive noise reduction, adaptive residual processing, and the Kalman filtering algorithm. We illustrate the positioning accuracy in Figure 8 and Table 3; the y-axis positioning accuracy of the three methods is of the same level.

From Figure 8 and Table 3, we can see that the proposed positioning system based on Kalman filtering has the highest accuracy among the three methods. More specifically, relative to adaptive filtering, the proposed method can improve the positioning accuracy by 81.57%. Especially when the SNR is below 20, the improvement is very obvious. Relative to the adaptive residuals method, the proposed method can improve the positioning accuracy by 9.34%; this is primarily due to the localization method proposed in this study, treating the minimum mean-square error rule as the best estimation criteria for signal noise reduction and adopting the Kalman filter to minimize the impact of noise.

### 4.4. Positioning Accuracy Evaluation with a Quaternary Array

**Figure 9:**
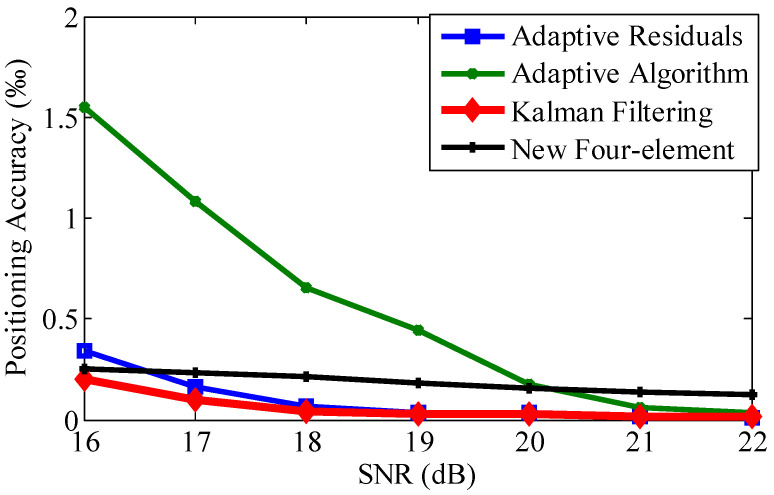
Comparison of the positioning accuracy of different methods with different SNR values.

In this section, we show the quaternary element array in Figure 3. We deployed Nodes 2–4 at the vertices of the right-angled triangle. Here, *L* = 8 *d* = 320 mm. We placed Node 1 at the right-angle sub-line. The distance between Node 1 and Node 3 is *d*. We adopted the three processing methods to perform the positioning. We show the statistical positioning accuracy in Figure 9 and Table 4, and Monte Carlo method is utilized to carry out 500 independent statistical calculations to find the positioning accuracy of various methods with different SNR values. We illustrate the positioning accuracy with different SNR values in Figure 9. We compare it with the other three related methods.

Figure 9 and Table 4 illustrate that the proposed positioning method improves the accuracy effectively compared with the other positioning methods. More specifically, relative to the adaptive filtering method, the proposed method can improve the accuracy by 82.49%. The proposed method can improve the accuracy by 16.14% compared with the adaptive residuals-based method, especially when the SNR is below 20 dB. Relative to the new four-element positioning method, the proposed positioning method can improve the accuracy by 70.97%. That is because, with the quaternary element array and Kalman filtering strategy, we can improve the positioning accuracy further.

### 4.5. Positioning Efficiency Evaluation

We evaluated the positioning efficiency in terms of positioning processing time and compared it with the other three methods; i.e., the adaptive residuals-based method, the adaptive algorithm-based method, and the new four-element array method. We illustrate the positioning processing time in Table 5.

Table 5 illustrates that the positioning processing time of the adaptive filtering algorithm and the new four-element positioning method is lower than that of the adaptive residuals algorithm and Kalman filtering positioning method. There are some filtering processing steps in the adaptive residuals algorithm-based positioning method and the Kalman filtering method; its processing time is higher, so the positioning improvement is at the cost of processing efficiency. We should determine the suitable positioning method according to the requirement of an application system. If we consider the positioning accuracy and efficiency comprehensively, the Kalman filtering method is applicable for many applications.

### 4.6. Discussion

#### 4.6.1. Discussion on Array Type

**Figure 10:**
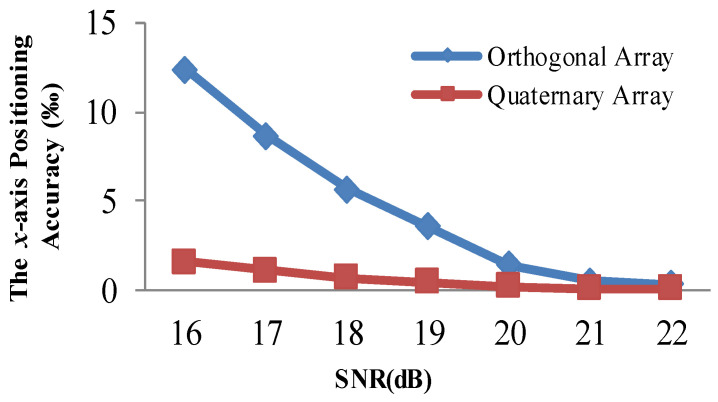
Comparison of the x-axis positioning accuracy of the adaptive residuals for different arrays and different SNR values.

**Figure 11:**
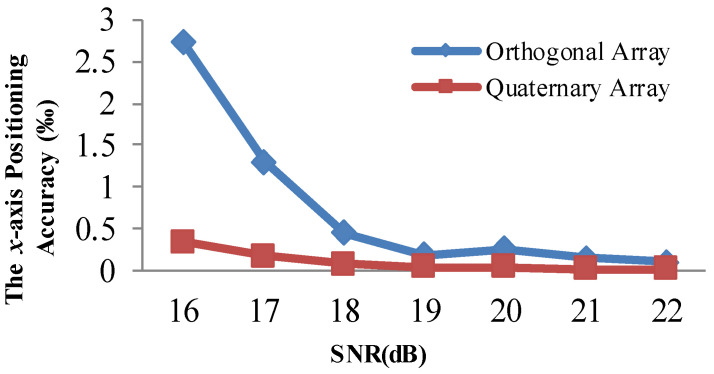
Comparison of the x-axis positioning accuracy of the adaptive algorithm for different arrays and different SNR values.

**Figure 12:**
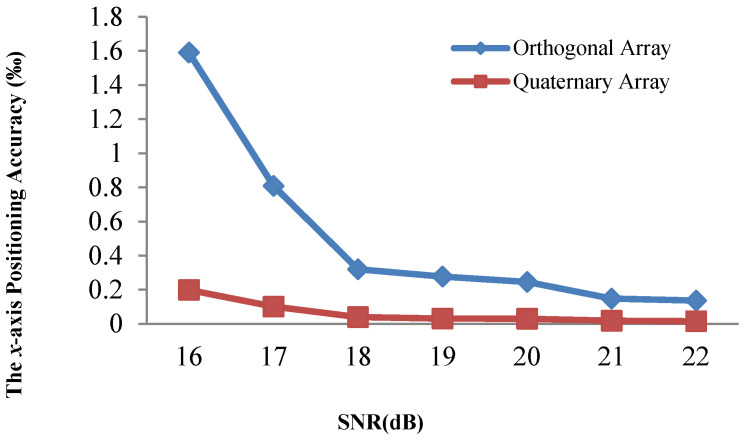
Comparison of the x-axis positioning accuracy of Kalman filtering for different arrays and different SNR values.

Figure 8 and Figure 9 demonstrate that the array type has a large effect on the acoustic positioning accuracy. We show the positioning accuracy of different works with the quaternary array and traditional array in Figure 10, Figure 11 and Figure 12.

Figure 10, Figure 11 and Figure 12 illustrate that the *x*-axis positioning accuracy with the quaternary array is higher than that with the traditional array. More specifically, their accuracy with the quaternary array is higher than that with the traditional array, 7.98-fold, 6.54-fold, and 7.99-fold under these three positioning methods.

**Figure 13:**
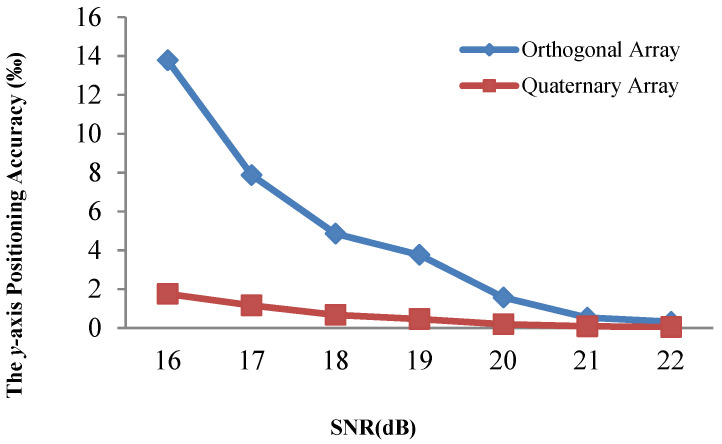
Comparison of the y-axis positioning accuracy of the adaptive residuals for different arrays and different SNR values.

**Figure 14:**
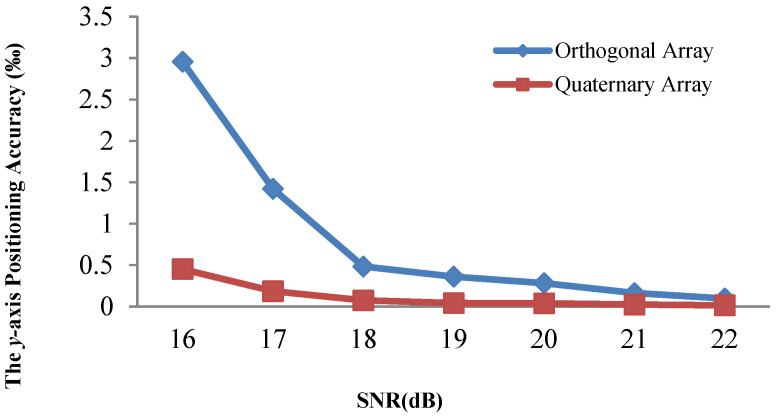
Comparison of the *y*-axis positioning accuracy of the adaptive algorithm for different arrays and different SNR values.

**Figure 15:**
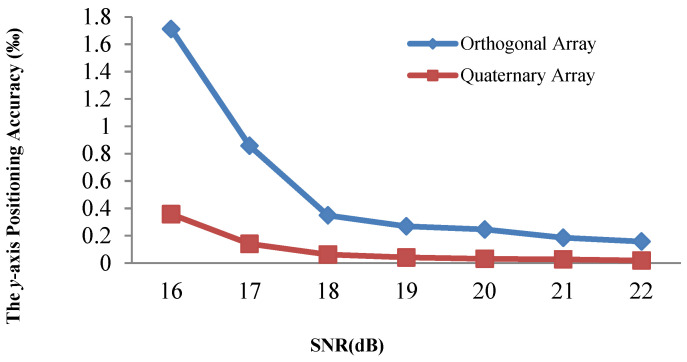
Comparison of the y-axis positioning accuracy of the Kalman filtering for different arrays and different SNR values.

We performed the same comparison for the *y*-axis, as shown in Figure 13, Figure 14 and Figure 15. The positioning results illustrate that the *y*-axis positioning accuracy with the quaternary array is higher than that with the traditional array. More specifically, the accuracy with the quaternary array is higher than that with the traditional array, 6.38-fold, 6.47-fold, and 6.84-fold, respectively; this is mainly because the quaternary array adopts a long size, which could reduce the negative effect of noise on the position accuracy. From Figure 10, Figure 11, Figure 12, Figure 13, Figure 14 and Figure 15, we can conclude that the quaternary array is a more suitable array when the acoustic positioning system is under deployment.

#### 4.6.2. Discussion on the Positioning Accuracy of Different Methods

Figure 8 and Figure 10 demonstrate that the proposed positioning method could gain a higher accuracy regardless of the kind of array. More specifically, with the traditional array, compared with the adaptive residuals and the adaptive algorithm positioning method, the proposed positioning method can improve the accuracy by 77.03% and 20.86% on average, respectively. With the quaternary array, compared with the adaptive residuals and the adaptive algorithm positioning method, the proposed positioning method could improve the accuracy by 81.57% and 1.75% on average, respectively; this is mainly owed to the Kalman filtering of the acoustic signals. We can obtain accurate phase difference information for a higher accuracy of the positioning results. The experimental results indicate the feasibility and validation of the proposed positioning method.

#### 4.6.3. Discussion on the Generalization of the Proposed Method

As there is no special requirement or equipment in our proposed underwater positioning method, we can apply it to any underwater positioning system with USBL. It can also be applied to a multiple-target underwater positioning system. In this case, we should utilize different frequencies to distinguish the response signals from the different targets.

## 5. Conclusions

During USBL positioning, for the low positioning accuracy problem caused by considerable noise interference caused by many negative factors, in this paper, the USBL positioning system based on the phase difference obtained by the Kalman filter algorithm is proposed. We first selected the non-equidistant, quaternary array to obtain the signal transmitted by the target. We then explored the Kalman filtering algorithm to achieve the high-precision phase difference information. Finally, we calculated the position of the target by bringing the phase difference information into the system model. The simulation results show that the underwater positioning method proposed in this paper can effectively improve the positioning error with a high positioning accuracy compared with the adaptive filtering algorithm and the adaptive filtering residual method; that is mainly due to our consideration of possible errors in all aspects of the positioning process. We propose the USBL positioning method based on the Kalman filter algorithm to obtain the phase difference and to achieve a high precision underwater target positioning, based on the phase difference acquisition mechanism and the minimum mean square-error as the best estimation criterion.

In the following research, we will implement and evaluate the performance of our proposed USBL positioning system. More specifically, we will cooperate with professional acoustic institutes and corporations to develop the hardware, the software, and the integration of the USBL positioning system. We will also evaluate it in a real application environment. It will take more than one year to accomplish this part of the research, and we will undertake delicate experiments to show the feasibility and validation of our proposed USBL positioning system.

## Figures and Tables

**Table 1:** Simulation conditions and parameters settings.

Symbol	Quantity	Value or Means
*R*	Target slant distance	3000 m
*c*	Acoustic velocity	1500 m/s
*d*	Adjacent array spacing	40 mm < 0.5 λ
*L*	Maximum array spacing	8 d = 320 mm
*f*_0_	Transponder frequency	1.35 kHz
*fs*	Sampling frequency	2000 kHz
*Tw*	Pulse width	5 ms
*SNR*	Signal-to-noise ratio (SNR)	20 dB

**Table 2:** Simulation conditions and parameters settings.

Symbol	Means	Setting
*f*_0_	Transponder frequency	1.35 kHz
*fs*	Sampling frequency	2000 kHz
SNR	Signal-to-noise ratio	16 dB–22 dB
*d*	Element distance	10 mm

**Table 3:** The positioning accuracy of the three methods with the traditional array.

SNR (dB)	16	17	18	19	20	21	22	Improvement
Adaptive Algorithm	12.3937	8.6505	5.6543	3.5464	1.3875	0.4869	0.3025	81.57%
Adaptive Residuals	2.7354	1.3014	0.4495	0.2836	0.2578	0.1453	0.0922	9.34%
Kalman Filtering	1.5905	0.8085	0.3197	0.2768	0.2456	0.1483	0.1367	-

**Table 4:** The positioning accuracy of the four methods with a quaternary array.

SNR (dB)	16	17	18	19	20	21	22	Improvement
Adaptive Algorithm	1.5492	1.0813	0.6569	0.4433	0.1734	0.0609	0.0378	82.49%
Adaptive Residuals	0.3419	0.1627	0.0687	0.0367	0.0322	0.0182	0.0115	16.14%
New Four-Element	0.2502	0.2334	0.2127	0.1850	0.1537	0.1355	0.1242	70.97%
Kalman Filtering	0.1988	0.1011	0.0400	0.0308	0.0295	0.0185	0.0151	-

**Table 5:** The positioning processing time of the three methods.

Positioning Method	Adaptive Residuals	Adaptive Algorithm	New Four-Element	Kalman Filtering
Positioning time (s)	552	11	1.2	48

## Abbreviations

LBL	Long Baseline
SBL	Short Baseline
USBL	Ultra-Short Baseline
SDN	Software-Defined Networking
AUVs	Autonomous Underwater Vehicles
UWNs	Underwater Wireless Networks
DVL	Doppler Velocity Log
